# Type 2 diabetes and depressive symptoms in the adult population in Mexico: a syndemic approach based on National Health and Nutrition Survey

**DOI:** 10.1186/s12889-022-14405-0

**Published:** 2022-11-09

**Authors:** Marcela Agudelo-Botero, Liliana Giraldo-Rodríguez, Claudio A. Dávila-Cervantes

**Affiliations:** 1 Centro de Investigación en Políticas, Población y Salud, Facultad de Medicina, Universidad Nacional Autónoma de México, Mexico City, Mexico; 2 Instituto Nacional de Geriatría, Mexico City, Mexico; 3 Facultad Latinoamericana de Ciencias Sociales (FLACSO), Sede México, Mexico City, Mexico

**Keywords:** Type 2 diabetes, Depressive symptoms, Violence, Obesity, Syndemic, Mexico

## Abstract

**Background:**

The syndemic approach allows the analysis of clusters of diseases that affect a population in contexts of geographic, social and economic inequalities at the same moment and time. This study aims to analyze, from a syndemic perspective, the relationship between type 2 diabetes (T2D) and depressive symptoms in Mexican adults and its association with individual, contextual and structural factors.

**Methods:**

Observational, cross-sectional study based on secondary data from Mexico’s National Health and Nutrition Survey 2018–19. The sample of this study consisted of 16 835 adults, which represented a total of 78 463 734 persons aged ≥ 20 years. Bivariate descriptive analyses were performed and logistic regression models were estimated to analyze the association between T2D and depressive symptoms with various co-variables. In addition, interactions between T2D and depressive symptoms with obesity, educational level, and socioeconomic status were tested.

**Results:**

In the study population, 12.2% of adults aged 20 years and older self-reported having T2D, 14.7% had depressive symptoms and 2.8% had both diseases. There was a statistically significant relationship between T2D and depressive symptoms. The prevalence of T2D and depressive symptoms was higher compared to people who did not have these two conditions. Obesity increased the probability of having T2D, while violence was statistically associated with people having depressive symptoms. A low level of education increased the odds ratio of having T2D and depressive symptoms.

**Conclusion:**

The availability of analytical frameworks such as the syndemic perspective could help to identify areas of opportunity for decision making and actions for population groups that–because of their individual, contextual and structural disadvantages–are at greater risk of experiencing poorer health outcomes due to the presence of T2D and depressive symptoms.

## Background

Syndemic concept refers to a framework that allows the analysis of clusters of diseases that affect a population in contexts of geographic, social and economic inequalities [1–3]. From this perspective, the aim is to understand how the co-existence of certain diseases of very diverse etiology can be influenced by specific environmental conditions, resulting in worse health outcomes. Therefore, the syndemic perspective contributes to understand diseases–and their interactions–in the light of structural determinants, including public policies, violence, educational backwardness, poverty, among others [1–4]. It is based on the idea that social and health phenomena do not occur in isolation, but rather that there is a reciprocal influence that leads to the exacerbation of the problems arising in these two aspects [1, 2, 4–8].

One of the syndemic clusters that has received the most attention globally is that of type 2 diabetes (T2D) and depression [9–12]. However, most of the available evidence comes from high-income countries and, to a lesser extent, from middle- and low-income countries [10, 13, 14]. From an epidemiological point of view, it has been found that people with T2D have an increased risk of suffering from depression; in turn, depression has an impact on the development and evolution of T2D (bidirectional causality) [15, 16]. Both diseases have common pathophysiological mechanisms, such as stress, sleep disorders, hypothalamic–pituitary–adrenal (HPA) axis dysfunction, inflammation, among others [15, 17], making these diseases major clinical challenges [18].

The literature has also identified some variables, which serve as factors associated with T2D and depression that have disproportionate effects in certain population groups, among which obesity, violence and poverty stand out [9, 12, 19–22]. Thus, people with lower incomes, from marginalized areas with high levels of obesity and violence, have a higher prevalence of T2D and depression, as well as higher morbidity and mortality due to these causes [12, 20, 23–25].

In Mexico, T2D has one of the highest burdens of disease in the adult population, as well as depression [23–27]. In 2019, the standardized rate of disability-adjusted life years (DALY) per 100,000 inhabitants was 2 261.1 for T2D and 630.6 for depressive disorders [28]. Of the total healthy years of life lost due to non-communicable causes in the country [25 469 089.6] in 2019, 13% were due to these two conditions [28]. On the other hand, Mexico has one of the highest prevalences of overweight and obesity in the world: seven out of 10 adults aged 20 years or older have one of these conditions [29, 30], and of the total number of adults with obesity, 3.6% suffer from the most severe form of obesity (body mass index [BMI] ≥ 40 kg/m^2^) [30, 31]. According to The Organization for Economic Co-operation and Development (OECD), Mexicans lose an average of 4.2 years of life due to overweight and obesity [32].

Regarding the social situation, Mexico has one of the highest homicide rates in the world, with a rate of 28.8 per 100,000 inhabitants in 2020 [33]. The overall perception of security is also critical since 65.8% of adults aged 18 years and older living in urban areas of the country believed that living in their cities was insecure by 2021 [34]. Likewise, 6.1% of women aged 15 years and older, who were surveyed in 2016, indicated having experienced violence at some point in their lives, while 39.8% suffered violence in the last year [35]. On the other hand, 43.9% of Mexicans lived in poverty, 8.5% in extreme poverty and 52.8% had an income below the income poverty line (2.9 percentage points more than in 2018) in 2020 [36]. However, there is a wide heterogeneity in the overall living conditions within the country that reinforces inequalities between regions [37, 38].

The previously described scenario provides the setting to understand T2D and depression from a syndemic perspective, as they are two frequent causes of disease in the Mexican population. The latter, in turn, is immersed in a diverse and complex social and economic reality, characterized by high rates of violence, poverty and diabetogenic risk. This paper aims to analyze, from a syndemic perspective, the relationship between T2D and depressive symptoms in Mexican adults and its association with individual, contextual and structural factors.

## Methods

### Study population and data

An observational, cross-sectional study was conducted based on secondary data from Mexico’s National Health and Nutrition Survey 2018–19 (ENSANUT, for its acronym in Spanish). This survey aimed to update the frequency, distribution, and trend of health and nutrition conditions and their social determinants, as well as to study the coverage, targeting, perceived quality, and user satisfaction of health and nutrition programs and services [39]. It is a national probabilistic survey that provides information at the national level, by federal states, for urban and rural areas, and by socioeconomic status [39]. The sample size was 50 654 dwellings at the national level, distributed in the 32 Mexican states [39]. For this paper, the sub-sample consisted of 16 835 adults, which represented a total of 78 463 734 Mexicans aged ≥ 20 years. This sub-sample was considered, given that the anthropometric tests (height and weight) for the estimation of people with obesity at the time of the survey were only taken from a fraction of the total adult sample.

### Measurements

The two diseases that were analyzed refer to cases of self-reported medical diagnosis of T2D and depressive symptoms. The first was based on the positive response to the question “Has any doctor told you that you have diabetes (or high blood sugar)?” The second was based on the scale of the Center for Epidemiological Studies Depression (CESD-7) [27], to assess depressive symptoms in the last week by means of seven questions with four response options: “rarely or never (less than one day)”, “few times or sometimes (1 to 2 days)”, “a considerable number of times (3 to 4 days)” and “all the time or most of the time [5 to 7 days]”. Each question was scored in a range from 0 to 3. The total score ranged from 0 to 21, where a higher score represented a greater degree of depressive symptoms. A cut-off value of nine points or more was set to indicate moderate or severe depressive symptomatology.

Co-variables were organized into three categories: individual, contextual and structural. The individual variables taken into account were: sex (female, male); age groups (20–34, 35–59, 60 and older), marital status (no married [single, divorced, widowed), married (married, common-law]) and economically active population (EAP) (yes [comprised by those persons who worked or who, although they did not work, actively sought to do so], no [those who did not work and who did not seek to do so]). Contextual variables included obesity and violence. Obesity was obtained from the body mass index (BMI), which was calculated as weight in kilograms divided by the square of height in meters (kg/m^2^). A score ≥ 30 kg/m^2^ was classified as obesity, according to criteria of World Health Organization (WHO), as previously described [30]. As for violence, it was related to the affirmative answer to the question “In the last 12 months, have you suffered any damage to your health due to robbery, aggression or violence?” (no, yes). Structural variables were defined as educational level (elementary school or less, middle school, high school, college or more) and socioeconomic status; this was created using information on housing construction material, the number of household appliances and the number of electrical appliances [30, 39]. The principal components method, generated from a polychoric correlation matrix, was used to create the index [39]. This procedure enabled the generation of an index that was subsequently categorized into four groups (low, medium low, medium high and high).

### Analysis

First, a descriptive analysis was performed to determine the general characteristics of the study population. Second, a bivariate analysis was carried out to identify relationships between individual, contextual and structural characteristics with T2D and depressive symptoms using *Pearson's chi-squared test* of independence from contingency tables. Third, two binomial logistic regression models were estimated to calculate the association between variables with T2D and depressive symptoms. Additionally, three interaction terms were calculated in each regression: 1) T2D or depressive symptoms * obesity, 2) T2D or depressive symptoms * educational level and, 3) T2D or depressive symptoms * socioeconomic status. These interactions were used to test whether the association of T2D with depressive symptoms–or the association of depressive symptoms with T2D–differ according to these characteristics. To show these associations, the results are presented as odds ratios (OR). Models were evaluated using the *Hosmer–Lemeshow* goodness-of-fit test. In all cases, a significance level *p* < 0.1 and 95% confidence intervals (CI) were used. For all the analyzes, the effect of the complex sampling design of the anthropometry section of ENSANUT was considered, taking the survey (*svy*) module of Stata®17 (Stata Corp, College Station, Texas, USA) [40].

## Results

Table 1 shows the characteristics of the study sample, as well as the prevalence of the diseases analyzed. Of the Mexicans aged ≥ 20 years [n = 16 835], 58 out of every 100 belonged to the female group, the highest proportion was in the age group of 35 to 59 years, 64.8% were married and nearly two thirds were EAP. Likewise, 36.3% of adults lived with obesity, while 2% had suffered harm in the last 12 months due to robbery, aggression or violence. Regarding structural variables, 64% had completed elementary school or less or middle school, and slightly more than half came from the medium–low socioeconomic status. Approximately, twelve and fifteen out of 100 adults reported a medical diagnosis of T2D and depressive symptomatology, respectively. Only 2.8% reported having both diseases.

**Table 1:** Description of the study population [*n* = 16 835]

Variables/Categories			n (Thousands)	%
**Diseases**	Tipe 2 diabetes*			
		No	14.7	87.8
		Yes	2.0	12.2
	**Depressive symptoms**			
		No	14.3	85.3
		Yes	2.5	14.7
**Individual variables**	**Sex**			
		Men	7.0	42.0
		Women	9.7	58.0
	**Age group**			
		20–34	5.3	31.4
		35–59	7.8	46.6
		60 +	3.7	22.0
	**Marital status**			
		No married	5.9	35.2
		Married	10.8	64.8
	**Economically active population**			
		No	6.4	38.0
		Yes	10.4	62.0
**Contextual variables**	**Obesity**			
		No	10.7	63.7
		Yes	6.1	36.3
	Violence**			
		No	16.3	98.0
		Yes	0.3	2.0
**Structural variables**	**Educational level**			
		Elementary or less	5.7	34.0
		Middle school	5.0	30.0
		High school	3.2	19.3
		College or more	2.8	16.7
	**Socioeconomic status**			
		Low	3.0	18.0
		Medium–low	8.4	50.0
		Medium–high	3.7	21.9
		High	1.7	10.2

^*^ *n* = 16 809. Cases of gestational diabetes were excluded

^**^ *n* = 16 775. Missing data was “no response”

The results of the bivariate analysis are shown in Table 2. Both diseases were found to be associated with each other, as were the rest of the variables analyzed (with the exception of violence, which was not statistically associated with T2D). It is noteworthy that the prevalence of in people with depressive symptoms was higher compared to those without symptoms (13.5% *versus* 23.1%); in turn, the percentage of adults with depressive symptoms was higher in the group with T2D (19.2%) respect to those without this disease (11%). As for obesity, the prevalence was higher in people with both T2D (45%) and depressive symptoms (40.5%); however, the percentage of incidents of violence was similar in adults with and without T2D, but notably higher for those with depressive symptoms. Both disease (T2D and depressive symptoms) were more frequent in the age groups of 35 to 59 years, as well as among women. For married people, the prevalence of T2D was 68.2% and 64.8% of depressive symptoms. It should be noted that this prevalence decreased as the level of schooling increased, for people who had either of the two diseases. In addition, the EAP had the highest percentage of adults with both diseases. Around 68% of people with T2D and 74% with depressive symptoms belonged to a low and medium–low socioeconomic status.

**Table 2:** Bivariate analysis between type 2 diabetes and depressive symptoms with individual, contextual and structural variables [*n* = 16 835]

Variables/Categories			%				
			**Type 2 diabetes**		**Depressive symptoms**		**Total**
			**No**	**Yes**	**No**	**Yes**	
**Diseases**	Type 2 diabetes*						
		No	-	-	89.0	80.8	87.8
		Yes	-	-	11.0	19.2	12.2
	Depressive symptoms*						
		No	86.5	76.9	-	-	84.8
		Yes	13.5	23.1	-	-	15.2
**Individual** **variables**	Sex*						
		Men	42.9	35.8	44.6	27.1	42.0
		Women	57.1	64.2	55.5	72.9	58.0
	Age group*						
		20–34	35.2	3.9	33.0	21.7	31.4
		35–59	46.4	48.1	46.1	49.4	46.6
		60 +	18.5	48.0	20.9	28.9	22.0
	**Marital statusξ**						
		No married	35.7	31.9	34.5	39.4	35.2
		Married	64.3	68.2	65.5	60.6	64.8
	Economically active population*						
		No	35.9	53.4	36.6	46.3	38.0
		Yes	64.1	46.6	63.4	53.7	62.0
**Contextual variables**	Obesity*						
		No	64.9	55.0	64.4	59.5	63.7
		Yes	35.1	45.0	35.6	40.5	36.3
	**Violenceξ**						
		No	97.9	98.3	98.4	95.6	98.0
		Yes	2.1	1.7	1.6	4.4	2.0
**Structural variables**	Educational level*						
		Elementary or less	30.9	56.2	31.7	47.2	34.0
		Middle school	30.7	25.0	29.8	31.3	30.0
		High school	20.6	10.2	20.3	13.5	19.3
		College or more	17.7	8.6	18.1	8.1	16.7
	Socioeconomic status*						
		Low	18.5	13.9	17.9	18.0	18.0
		Medium–low	49.5	53.4	49.0	55.9	50.0
		Medium–high	21.7	23.2	22.4	18.6	21.9
		High	10.3	9.5	10.7	7.5	10.2

^*^ Significance of the Chi-square test *p* < 0.01 in both variables [type 2 diabetes and depressive symptoms]

^ξ^ Significance of the Chi-square test *p* < 0.01 only for depressive symptoms

This test indicates that the variable has a statistically significant association with diabetes or depressive symptoms

In model 1 of the logistic regression (Table 3), it can be observed that having depressive symptoms increased the possibility of living with T2D (OR = 1.33; *p* < *0.01*), compared to those who did not have T2D. Being 35–39 years of age and 60 years or older also considerably increased the OR of T2D; while belonging to the EAP and having middle-school or more, decreased them. Obesity increased 38% (*p* < *0.01*) the possibility of having T2D. In model 2 (Table 3), T2D increased the possibility of an adult having depressive symptoms by 39% (*p* < *0.05*), and the same trend was observed for people in the age group 35–39 and 60 years or older. Obesity was not associated with depressive symptoms, but violence was associated with depressive symptoms (OR = 3.45; *p* < *0.01*). Likewise, being married, having middle-school or more, and having a high or medium–high socioeconomic status reduced the OR of depressive symptoms. Finally, none of the interactions estimated in models (1 and 2) were statistically associated with T2D or depressive symptoms.

**Table 3:** Logistic regression models between type 2 diabetes and depressive symptoms with individual, contextual and structural variables

Variables/Categories		Model 1:		Model 2:	
		**Type 2 diabetes**		**Depressive symptoms**	
		**OR**	**[95% Cl]**	**OR**	**[95% CI]**
**Disease**		***n*** = 16,809		***n*** = 16,750	
**Type 2 diabetes**	No +	-	-	1	-
	Yes	-	-	1.33*	[1.00,1.79]
**Depressive symptoms**	No +	1	-	-	-
	Yes	1.39**	[1.03,1.88]	-	-
**Individual variables**					
**Sex**	Men +	1	-	1	-
	Women	1.16	[0.96,1.41]	2.06***	[1.74,2.45]
**Age group**	20–34 +	1	-	1	-
	35–59	7.90***	[5.67,10.89]	1.51***	[1.24,1.83]
	60 +	16.80***	[11.86,23.79]	1.55***	[1.23,1.96]
**Marital status**	No married +	1	-	1	-
	Married	1.16*	[0.98,1.38]	0.78***	[0.66,0.91]
**Economically active population**	No +	1	-	1	-
	Yes	0.76***	[0.63,0.92]	0.93	[0.80,1.10]
**Contextual variables**					
**Obesity**	No +	1	-	1	-
	Yes	1.38***	[1.13,1.70]	1.08	[0.91,1.27]
**Violence**	No +	-	-	1	-
	Yes	-	-	3.45***	[2.19,5.42]
**Structural variables**					
**Educational level**	Elementary or less +	1	-	1	-
	Middle school or more	0.68***	[0.55,0.86]	0.65***	[0.54,0.78]
**Socioeconomic status**	Low or Medium–low +	1	-	1	-
	High or Medium–high	1.04	[0.81,1.32]	0.73**	[0.57,0.92]
**Interactions**					
Type 2 diabetes*Obesity	No obesity/no Type 2 diabetes +	-	-	1	-
	Obesity/ Type 2 diabetes	-	-	1.12	[0.78,1.61]
Tipe 2 diabetes*Educational level	Elemenary or less/no Type 2 diabetes	-	-	1	-
	Middle school or more/ Type 2 diabetes	-	-	1.15	[0.79,1.69]
Type 2 diabetes*Socioeconomic status	Low or Medium–low/no Type 2 diabetes +	-	-	1	-
	High or Medium–high/ Type 2 diabetes	-	-	0.97	[0.6,1.57]
Depressive symptoms*Obesity	No obesity/no Depressive symptoms +	1	-	-	-
	Obesity/Depressive symptoms	0.99	[0.69,1.44]	-	-
Depressive symptoms*Educational level	Elemenary or less/no Depressive symptoms +	1	-	-	-
	Middle school or more/Depressive symptoms	1.26	[0.86,1.86]	-	-
Depressive symptoms*Socioeconomic status	Low or Medium–low/no Depressive symptoms +	1	-	-	-
	High or Medium–high/Depressive symptoms	0.87	[0.54,1.41]	-	-
***Constant***		0.02***	*[0.01, 0.03]*	0.06***	*[0.04, 0.09]*

+ Reference category; *** *p* < 0.01; ** *p* < 0.05; * *p* < 0.1

## Discussion

This study contributes to the parallel analysis of T2D and depressive symptoms and their syndemic relationship, analyzed through individual, contextual and structural characteristics. The results of this study support the idea that T2D and depressive symptoms are closely related, and that people in the most disadvantaged socioeconomic situations (lower educational level or lower socioeconomic strata) are more likely to have these diseases [10–12, 20, 23, 25]. In the particular case of Mexico, this conditions are also exacerbated by obesity (only for T2D), as well as by violence (only in the case of depressive symptoms). These results have been corroborated, at different times and populations, mainly through qualitative ethnographic studies [19, 41–43]. In this way, it has been demonstrated how the experiences of having these diseases are lived in a very particular way based on the specific social, economic and cultural structures that surround the individuals. Therefore, it has been suggested to use data and methods that are locally relevant in order to pinpoint syndemic synergies [11, 44].

Although T2D and depression have been previously analyzed from a syndemic perspective, the magnitude of the effects varies from one population to another, which depends significantly on the epidemiological profile and the social and economic development indicators of each country. The prevalence of T2D and depressive symptoms in Mexico was higher than those reported for Brazil, where the percentages were 6.2% and 7.9% (in adults aged 18 years and older), respectively [20]. In Mexico, according to ENSANUT 2018–19, 10.3% of adults aged ≥ 20 years self-reported T2D and 13.6% had depressive symptoms [39, 45]; specifically, in the subsample of this study, these percentages were 12.2% for T2D and 14.7% for depressive symptoms. However, far from changing this situation, a gradual deterioration in the health of the adult population has been evidenced because the prevalence of non-communicable diseases has been steadily rising in recent years (2006–2018/19) [39].

On the other hand, the results show that obesity and violence are highly relevant variables in understanding T2D and depression among the Mexican population. First, obesity, particularly severe (type III) obesity, has had the highest increase versus total obesity, as well as type-I and -II obesity. This means that, between 2000 and 2018–19, while overall obesity increased by 1.7% each year, severe obesity increased by 4.4% [30, 31]. Obesity has even been called a pandemic, identified as the leading cause of ill health worldwide [46]. Meanwhile, the unprecedented increase in homicides has resulted in a reversal of life expectancy among men and a slowdown for women [47, 48]. Although this is a phenomenon that was concentrated in certain regions and states until a few years ago, it has been progressively spreading throughout the country [49, 50]. Record highs in homicides have been reached and this is unlikely to change in the near future, as the authorities have failed to take action in the last decade.

Syndemia of T2D and depression is immersed in a diverging epidemiological transition [38] and a process of accelerated demographic aging. It represents a challenge of great magnitude for Mexico´s health system, which must face diseases of diverse nature that imply different approaches. This, considering that there is a fragmentation and segmentation in the provision of services and an inequitable distribution of human, financial and material resources that contributes to widening health disparities [51, 52]. Mexico, like other countries, is facing the overlapping of several epidemics, which have socioeconomic determinants as a common feature [53].

Previous studies have documented the importance of understanding T2D and depression in contrast to individual, social, environmental, economic, political and cultural conditions, as these are catalysts of inequities in health outcomes. For example, Diderichsen and Andersen have documented in Brazilian population that having a precarious position in the labor market and low levels of educational level, as well as belonging to low socioeconomic strata is associated with an increased risk of presenting both conditions, especially when they occur in interaction with obesity and violence [12, 20]. Therefore, addressing these conditions requires health interventions, as well as modifying the social causes underlying their etiology and perpetuating health inequalities [19].

Concurrence of T2D and depressive symptoms in Mexican older adults meets the three main premises of the syndemic model: 1) the high frequency of the diseases, 2) the relationship between both diseases, and 3) the convergence of social, economic and health factors that magnify the disparities. T2D and depression are not only interrelated by clinical or physiological aspects, as social and economic factors play a role in the occurrence and complications of these diseases [26].

## Limitations and strengths

This study has some limitations that should be mentioned. The first limitation concerns the data used, since they are cross-sectional data and do not allow to see the causality of the dependent variables and their potential syndemic effects over time. People´ life circumstances may have changed and this may affect the analyzed relationship between T2D and depressive symptoms among individuals. We were also unable to know exactly the sequence of the diseases, that is, the moment in which they appeared and the time of exposure to them. Future longitudinal studies could help to better understand these aspects.

Another limitation is related to the fact that the information on the different variables of interest in this study is self-reported, which could mean a bias that may lead to underestimation of the data. On the other hand, the scale used to measure “depressive symptoms” only refers to the last seven days, prior to the survey, so it is not possible to establish whether this reflects a temporary situation or a more prolonged clinical condition (major depression). In addition, due to the same characteristics of the study, it was not possible to establish the bidirectional relationship between underdiagnosed T2D, since this information is not available in the survey used. In this regard, a study conducted by McGrath et al. (2021), found discrepancies in the relationship between T2D and undiagnosed depression in adults aged 50 years and over in Ireland, England and the USA, for which they recommend improving the detection of depression among older adults, especially those living with T2D [54]. On the other hand, Habib et al. (2022), considering it necessary to carry out simultaneous diagnoses and treatments of both diseases, in order to reduce complications and the burden on health [55].

Finally, the adaptation of ENSANUT data to determine the syndemic relationships was a limitation we faced; this source of information lacks some variables that are key to understanding the complex connections between health and living conditions of individuals. It is worth noting that the syndemic perspective comes from medical anthropology and most studies have been based on qualitative techniques. However, there has been a growing interest in conducting quantitative studies in recent years that also reflect the synergistic interactions between comorbidities with social and economic factors. This paper is an effort to address these issues.

A strength to highlight is that the results are broad in scope since they are applicable to adult population aged 20 years or older. It was also the first study that performed a joint analysis of T2D and depressive symptoms based on a nationally survey.

## Conclusion

The availability of analytical frameworks such as the syndemic perspective could help to identify areas of opportunity for decision making and actions for population groups that, because of their individual, contextual and structural disadvantages, are at greater risk of experiencing poorer health outcomes due to the presence of T2D and depressive symptoms. Similarly, evidence generated can support the comprehensive monitoring of the Sustainable Development Goals (SDG). Prior to the SARS-CoV-2 pandemic (COVID-19), significant lags were already noted in these SDG in the poorest states of the country [37]. The COVID-19 pandemic has compromised the achievement of the SDG, especially in middle- and low-income countries, where macro-economic conditions have worsened [56]. Hence, public health actions should be supported by multi-sectoral and comprehensive policies where the individual, contextual and structural factors converge.

## Data Availability

ENSANUT 2018–19 data files and documentation are of local use and available at https://ensanut.insp.mx/encuestas/ensanut2018/index.php#:~:text=La%20ENSANUT%202018%20tiene%20como,determinantes%20sociales%20en%20el%20pa%C3%ADs. All data used and/or analyzed during the current study are available from the corresponding author upon reasonable request (claudio.davila@flacso.edu.mx).

## Abbreviations

BMI: Body mass index
CESD-7: Center for Epidemiological Studies Depression
CI: Confidence intervals
DALY: Disability-adjusted life years
EAP: Economically active population
ENSANUT: Encuesta Nacional de Salud y Nutrición
HPA: Hypothalamic–pituitary–adrenal
OECD: The Organisation for Economic Co-operation and Development
OR: Odds ratios
SARS-CoV-2: COVID-19
SDG: Sustainable Development Goals
T2D: Diabetes type 2
WHO: World Health Organization
